# 3D Cell Culture Models as Recapitulators of the Tumor Microenvironment for the Screening of Anti-Cancer Drugs

**DOI:** 10.3390/cancers14010190

**Published:** 2021-12-31

**Authors:** Mélanie A. G. Barbosa, Cristina P. R. Xavier, Rúben F. Pereira, Vilma Petrikaitė, M. Helena Vasconcelos

**Affiliations:** 1 Cancer Drug Resistance Group, IPATIMUP—Institute of Molecular Pathology and Immunology, University of Porto, 4200-135 Porto, Portugal; melanieb@ipatimup.pt (M.A.G.B.); cristinax@ipatimup.pt (C.P.R.X.); 2 i3S—Instituto de Investigação e Inovação em Saúde, Universidade do Porto, 4200-135 Porto, Portugal; ruben.pereira@ineb.up.pt; 3 Biofabrication Group, INEB—Instituto de Engenharia Biomédica, Universidade do Porto, 4200-135 Porto, Portugal; 4 ICBAS—Instituto de Ciências Biomédicas Abel Salazar, Universidade do Porto, 4050-313 Porto, Portugal; 5 Laboratory of Drug Targets Histopathology, Institute of Cardiology, Lithuanian University of Health Sciences, A. Mickevičiaus g 9, LT-44307 Kaunas, Lithuania; vilmapetrikaite@gmail.com; 6 Institute of Biotechnology, Life Sciences Center, Vilnius University, Saulėtekio al. 7, LT-10257 Vilnius, Lithuania; 7 Department of Biological Sciences, FFUP—Faculty of Pharmacy of the University of Porto, 4050-313 Porto, Portugal

**Keywords:** 3D cell culture models, tumor microenvironment, cellular co-culture, stromal cells, preclinical assays

## Abstract

**Simple Summary:**

Three-dimensional (3D) cell culture models have been proposed as alternatives for initial drug screening to increase drug development efficiency. In addition to their ability to reproduce key aspects of the tumor architecture and microenvironment, 3D cell culture models may help decrease the use of laboratory animals in drug testing, in accordance with the 3R principles (Replacement, Reduction, Refinement). This review aims to help researchers making a transition from two-dimensional (2D) to 3D cell culture models for drug screening, by discussing the impact of 3D models on cancer research, their advantages, limitations, and compatibility with high-throughput screenings. It also outlines the relevance of available readouts provided by such models as well as the importance of incorporating key microenvironmental cues towards improving the predictive value of drug efficacy and safety.

**Abstract:**

Today, innovative three-dimensional (3D) cell culture models have been proposed as viable and biomimetic alternatives for initial drug screening, allowing the improvement of the efficiency of drug development. These models are gaining popularity, given their ability to reproduce key aspects of the tumor microenvironment, concerning the 3D tumor architecture as well as the interactions of tumor cells with the extracellular matrix and surrounding non-tumor cells. The development of accurate 3D models may become beneficial to decrease the use of laboratory animals in scientific research, in accordance with the European Union’s regulation on the 3R rule (Replacement, Reduction, Refinement). This review focuses on the impact of 3D cell culture models on cancer research, discussing their advantages, limitations, and compatibility with high-throughput screenings and automated systems. An insight is also given on the adequacy of the available readouts for the interpretation of the data obtained from the 3D cell culture models. Importantly, we also emphasize the need for the incorporation of additional and complementary microenvironment elements on the design of 3D cell culture models, towards improved predictive value of drug efficacy.

## 1. Introduction

The scientific community and the pharmaceutical industry have been investing in the development of novel antitumor drugs, which have to be evaluated using various in vitro and in vivo assays. These assays are essential for the preclinical screening of the drug development process, and support the transition of the best-performing compounds for human clinical trials [1]. Although the increase in drug development throughput and technological advances should have allowed a more reproducible and cost-effective development of successful therapies [2], only 5% of the new antitumor molecules successfully gain clinical approval, while the remaining percentage fail as a result of toxicity and poor efficacy [3,4,5]. Indeed, the development of effective, safe and economically viable antitumor drugs remains a major challenge. The poor clinical performance of new drugs is possibly explained by the low correlation of the preclinical in vitro and in vivo data with the results from the clinical trials [6]. Unfortunately, this is a consequence of the lack of disease-relevant preclinical models able to recreate the physiopathology of the tumor and recapitulate the tumor complexity regarding the matrix microenvironment as well as the interactions between tumor cells and the surrounding niche [7,8].

Over the past years, three-dimensional (3D) cell models have gained attention for their ability to more closely mimic the features of tumors in vivo, bridging the gap between two-dimensional (2D) cell culture systems and in vivo models [9,10]. In addition, 3D models might present an alternative to the use of animals in biomedical research, thus respecting the 3R principles imposed by ethical and regulatory laws [10,11].

In this review, we summarize the 3D tumor models available for drug screening, the challenges and limitations of these models, and the most adequate readouts and their potential to extract predictive drug response data.

## 2. Pros and Cons of the Current Models for Anti-Cancer Drug Testing

### 2.1. 2D Cell Culture as the Basis of Preclinical Studies

In the preclinical development phase of the classic drug development pipeline, in vitro cell-based assays are mainly performed using 2D cell culture models, where immortalized cells are grown in a suspension or as monolayers on a flat surface, and those cells are then treated with a drug at a desired concentration [8].

The 2D cell culture models present several advantages over the 3D cell culture models, such as the simple implementation of high throughput screening assays, the level of standardization and reproducibility, and the simplified assay conditions along with the straightforward interpretation of the results [7,12]. However, 2D cell cultures do not replicate the complexity of the 3D tissue architecture nor the communication between tumor cells and the tumor microenvironment (TME) [12,13]. Indeed, solid tumors often grow under hypoxia conditions, present some cells with stem cell characteristics, and slow proliferation, among other features, that contribute to drug resistance and are not represented in monolayer cells [7]. Cells cultured in monolayers are exposed to surfaces with high stiffness, which alters the cells’ behavior, differentiation, gene expression, and drug sensitivity [14,15]. In fact, the biosynthesis of drug-metabolizing enzymes, which are essential in drug toxicity assays, is one of the first tissue-related functions to be impaired in monolayer cell cultures [10].

Therefore, 2D models also fail to reproduce the gradients in nutrients, molecules, and oxygen, which are commonly found in the TME and vary with the tumor’s size and mass [16]. Additionally, in order to maintain the normal cell growth and ensure the presence of necessary nutrients, monolayer cells must be trypsinized regularly, a process that may in the long-term originate genotypic and phenotypic alterations, thus influencing cells’ growth and response to external and internal stimuli [17,18].

Importantly, reproducing the phenotype of a tumor using in vitro cultured cells is indispensable to obtain more accurate biomedical data. The 2D cell cultures fail to mimic the tumor-specific architecture, the mechanical and biochemical signals, and the cell-cell and cell-extracellular matrix (ECM) communications [10]. Hence, the predictive value of assays performed in 2D cell culture is impaired and justifies the need for developing novel preclinical cell culture models for drug development, with better predictive outcomes. Indeed, this is of utmost importance to identify more effective and less toxic drugs, prior to the initiation of the clinical trials [10]. The improvement on drug development methodologies could also confer a substantial cost-effective advantage over the current drug development models [7].

### 2.2. In Vivo Studies as the Last Step of the Preclinical Studies towards Clinical Trials

According to industry standards, novel drugs must be tested in at least two species of animal models, usually a rodent and a non-rodent, before being admitted to human clinical trials [19]. However, tests in animal models are not as standardized as in 2D cell models, which can lead to unreliable drug testing results [20,21]. For example, when using in vivo studies, the choice of gender, the number of animals to enroll the study, and animal age, as well as the level of stress to which animals are exposed, vary between laboratories, and thus might have a significant impact on the experimental results [20]. The mouse model is by far the most frequently used in vivo model for several reasons: (1) low maintenance cost; (2) short gestation period; (3) easy model for genetic manipulation; and (4) ability to grow tumor cells from patients, forming patient-derived tumor xenografts (PDTX) for more personalized drugs testing [22].

Nevertheless, regarding the PDTX approach, the engraftment process, plus the maintenance of the PDTX and the molecular profiling required to evaluate if the tumor isolated from the mouse mimics the original patient tumor, are very expensive [21]. Additionally, considering the time required for this drug testing, the results may be obtained after the tumor of the patient suffered mutations or entered metastasis, thereby compromising the effectiveness of the treatment regime [21]. The in vivo studies also present some biological limitations that impair their efficacy and predictive value. For instance, the TME of the mouse is very different from the TME of the human, and although the co-implantation of both human tumor cells and stromal cells in animal models has been suggested, reports have demonstrated that human stromal cells are quickly replaced by the mouse stroma and immune cells [23]. In addition, animal experimentation is subjected to ethical and regulatory laws that imply a reduction in the number of tested animals and careful planning of the experiments and procedures in order to decrease animal distress [24]. The wrong reduction in the animal sample sizes, the use of poorly-validated animal models, and the application of inappropriate statistics have been pointed out as some of the main reasons underlying the poor scientific validity and reproducibility of the in vivo studies in biomedical research [25].

### 2.3. 3D Cell Culture Models as Recapitulators of Tumors In Vivo

The transition from 2D to 3D cell culture models is driven by the need to reduce drug failure during clinical trials [26]. The development of more sophisticated and reproducible 3D cell culture models allows to increase the predictive power of cell-based drug screenings and decrease the use of laboratory animals for testing drugs, which is in alignment with the principles of the 3Rs (Replacement, Reduction, Refinement) [10,11]. Moreover, 3D cell culture models confer specific advantages over in vivo models regarding the recapitulation of the human tumor-stromal crosstalk, by eliminating the existent cross-species incompatibilities of the PDTX models [23]. In addition, the properties of 3D cell culture models can be better managed and tuned, when compared to the biological complexity of in vivo models [27,28,29]. In contrast to the 2D cell culture, where cells grow at an unnaturally rapid pace, cells in 3D cultures proliferate at a rate that is more realistic and that can vary between the different techniques used and type of cells [15]. Consequently, 3D spheroid systems are suitable for studying the long-term effects of drugs, as the cells can remain functionally stable for several weeks [26,30].

3D cell culture models replicate the natural tumor architecture, thereby presenting an external proliferating zone, an internal quiescent zone with limited oxygen, nutrient and growth factor distribution, and a necrotic and hypoxic core [31,32]. All these factors might influence drug response. The spheroid size can be controlled through the cell seeding density, and its optimization is crucial for experiments requiring long-term spheroid monitoring, or when faced with specific assay limitations (e.g., limitations in the fluorescence staining dye penetration or in the instrument’s imaging capacity) or assay preferences (e.g., in the replication of the hypoxic core) [33].

Importantly, hypoxia is a phenomenon that promotes the development of aggressive tumor phenotypes, through activation of DNA (deoxyribonucleic acid) damage repair proteins, alteration in cellular metabolism, and decrease in proliferation, which consequently influences the tumor’s sensitivity to drugs [16,34]. Moreover, oxygen-deprived cells from the core of the spheroid can acidify the environment (e.g., through increased lactate production and increased carbonic anhydrase IX expression) [35,36,37], leading to a decrease in the cellular uptake of drugs (especially weak basic drugs such as doxorubicin, mitoxantrone, vincristine, vinblastine, anthraquinones, and vinca alkaloids), whose protonation in acidic environments impairs their ability to cross the cellular membrane [35]. For instance, in HCT116 colon cancer spheroids, the doxorubicin uptake decreased with the spheroid’s depth and the half-maximal inhibitory concentration (IC_50_) for this drug was higher at a lower extracellular pH (pH = 6.4), in accordance with the pH-partition theory [38]. In fact, hypoxic conditions have been reported in the core of spheroids larger than 200 μm in diameter, with the lack of oxygen in the center of spheroids explained by the increase in oxygen diffusion distances, as well as by the increase in oxygen consumption attributed to the higher proliferation of cells from the external region of the spheroid [33,39,40]. Even though the effect of hypoxia can be studied using monolayer cultures (placed in gas-controlled chambers), this 2D model still fails to recreate key aspects of the tumor biology that can impact the cells’ behavior and drug response (e.g., the stiffness of the surrounding environment, nutrient and oxygen gradients across the tumor, the cell spatial distribution, and specific cell-cell interactions) [16,41]. Therefore, 3D models are more suitable to test cellular drug response taking into consideration aspects that are impossible to consider in 2D cell culture models, such as oxygen and pH gradients. Moreover, as a consequence of the better representation of the real tumor, the gene and protein expression levels in 3D models are found to be more similar to those found when using in vivo models, when compared to the 2D models [15]. These characteristics make the 3D models particularly advantageous for the identification of new biomarkers of disease, which could in time enhance the discovery of more effective drugs [42]. Indeed, drugs developed around a biomarker-driven rationale are less likely to fail at late stages of drug development [4].

Moreover, 3D cell culture models can be used to increase the predictive value of nanomedicine screening, by modeling the selective penetration, accumulation, retention, and distribution of nanocarriers, thus providing important information regarding the drug behavior inside the tumor mass [43,44,45]. Surface functionalization of nanoparticles with specific molecules (e.g., PEGylated nanoparticles) has allowed the improvement of the penetration of drugs into specific areas (e.g., center or periphery layers) inside the spheroids [43,46]. Importantly, other factors such as size, shape, and surface charge of the nanoparticles have an impact on the penetration and retention of nanoparticles inside spheroids [47,48].

Despite the numerous advantages, 3D models also have downsides when compared to 2D cell models for drug screening. For example, it is difficult to visualize the typically highly scattered (and hundreds of micrometers thick) 3D samples on microscopes [10,39]. Analyzing 3D samples by flow cytometry can also be challenging, as it requires the dissociation of the spheroids into a single-cell suspension, usually by treating the cells with enzymes (or an enzyme cocktail) that promotes cellular detachment [6,49]. Moreover, after spheroids dissociation, distinguishing between the outer and inner cells becomes difficult and, consequently, important information could be lost due to this process [50]. One alternative to handle this downside is to mark the spheroids with fluorescent markers before the dissociation of these spheroids (e.g., markers of hypoxia could indicate the presence of cells localized at the core of the spheroid).

Other disadvantages of 3D cell culture models, when compared to 2D ones, are the higher cost of most of the techniques involved in creating the 3D models, especially for large-scale studies, as well as the significantly higher time required to be performed [39]. Other important disadvantages of using 3D models include the lack of affordable standard methods to develop 3D cell cultures, as well as of the right assays to test drugs with future clinical relevance [33], associated with the difficulty to replicate experiments and to interpret the resulting data [51].

**Figure 1:**
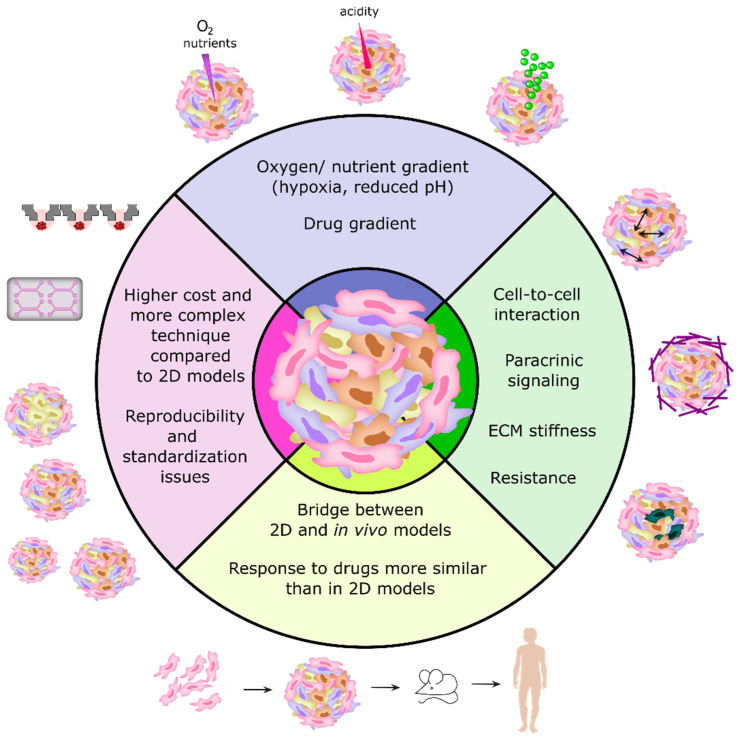
The main features and advantages/disadvantages of 3D cell models in comparison to 2D cell models (cells grown in monolayers).

Therefore, the use of 3D cancer models for preclinical drug screening can be challenging due to the large variabilities between the different models, and the difficulty in combining these models with high-throughput screening (HTS) and high-content imaging (HCI) approaches [18,26]. A summary of the main features of 3D cell culture models is represented in Figure 1. In the near future, the development of more standard protocols and methods for establishing 3D cultures, as well as more accurate quantitative analysis and 3D imaging techniques, will be necessary to make the most of the benefits of using 3D models in cancer research and antitumor drug screening [7,10].

## 3. 3D Cell Culture Models Available for Cancer Drug Screening

### 3.1. Classification of 3D Tumor Models

Despite the fast development in the field of 3D cell culture, the terminology used to classify 3D models and the techniques implemented have been used inconsistently throughout the literature [52]. In 2015, Weiswald et al. proposed a classification for the main 3D in vitro cancer models [52]. Under this classification, 3D tumor sphere models can be divided into four types, which differ in terms of culture methods and sphere biology: multicellular tumor spheroids (MCTS), tumorospheres, tissue-derived tumor spheres (TDTS), and organotypic multicellular spheres (OMS).

The MCTS contain mono- or heterotypic cell populations (e.g., co-culturing tumor cells with stromal cells, such as immune cells, endothelial cells and/or fibroblasts) and can be obtained by culturing the cells under non-adherent conditions [16,52]. Out of the four models, the MCTS model is the one that allows maximal control over factors that impact cell behavior, such as the influence from non-tumor cell types (only if the model contains monotypic populations) and the influence of the heterogeneous phenotype of tumor cells [16]. In addition, the MCTS model has a higher reproducibility and speed of spheroid generation [16]. The MCTS model containing only cancer cells is considered simplistic as it typically employs immortalized cell lines that, while being convenient for high-throughput screening, do not accurately represent a real tissue if not co-cultured with other cell types [33,53]. On the other hand, co-cultures enhance the complexity of the model, consequently affecting the throughput, and require optimization in terms of cell ratios and cell media components in order to allow the proper growth of both cell types [53].

Regarding tumorospheres, this model allows the expansion of the stem cell population into floating clusters, which are obtained through clonal expansion of a single cell suspension, under non-adherent conditions, in culture media supplemented with specific growth factors (“stem cell medium”) [16]. The tumorospheres can be formed from cell lines or tumor tissue, and in the case of tumor samples require a first step of mechanical and enzymatic dissociation to form the single-cell suspension [52].

Concerning TDTS, this model can be obtained by partial mechanical or enzymatic dissociation of tumor tissue, to separate primary cancer cells from non-tumor cell types while maintaining the cell-cell contact of cancer cells. This approach differs from the OMS model that is obtained by cutting the primary tumor tissues [52]. Both TDTS and OMS models recreate the tumor growth and expression profiles more accurately, when compared to MCTS and tumorospheres, but the OMS model provides additional complexity by enabling the presence of stromal cells [16].

The OMS model is therefore the most suitable 3D model for evaluating the therapeutic response of an individual’s tumor to a drug, being highly promising for personalized medicine [16,54]. This model also enables the study of rare subtypes of cancers for which there are no immortalized cell lines [54]. Nevertheless, the high cost associated with these organotypic models, and the limited availability and heterogeneity of the source material, impairs their use for in vitro drug screening [53]. Additionally, in order to promote 3D reorganization, OMS models also need highly specific media, supplements, and exogenous extracellular matrix preparations, becoming time-consuming to handle and a challenge for automation equipment and high-throughput screening [53].

### 3.2. Methodologies for Developing 3D Cell Culture Models

3D cell culture offers a panoply of methods that might answer specific questions and take into account specific cancer hallmarks. Importantly, each method provides its own advantages, limitations, and applications, which should be taken into consideration when selecting the method to use.

In general, 3D cell culture techniques can be divided into two types: scaffold-free and scaffold-based techniques [8]. Table 1 summarizes the techniques available for the development of 3D cell culture models for drug screening.

#### 3.2.1. 3D Scaffold-Free Culture Techniques

Scaffold-free techniques take advantage of the natural ability of many cell types to self-aggregate without requiring biomaterials, forming spheroids where cells secrete their own ECM over time [29]. These techniques are mostly divided into forced floating methods, the hanging drop method, and agitation-based approaches [8,10]. However, other scaffold-free techniques are available, such as the pellet culture method, the micromolding method, and the magnetic levitation/bioprinting.

**Figure 2:**
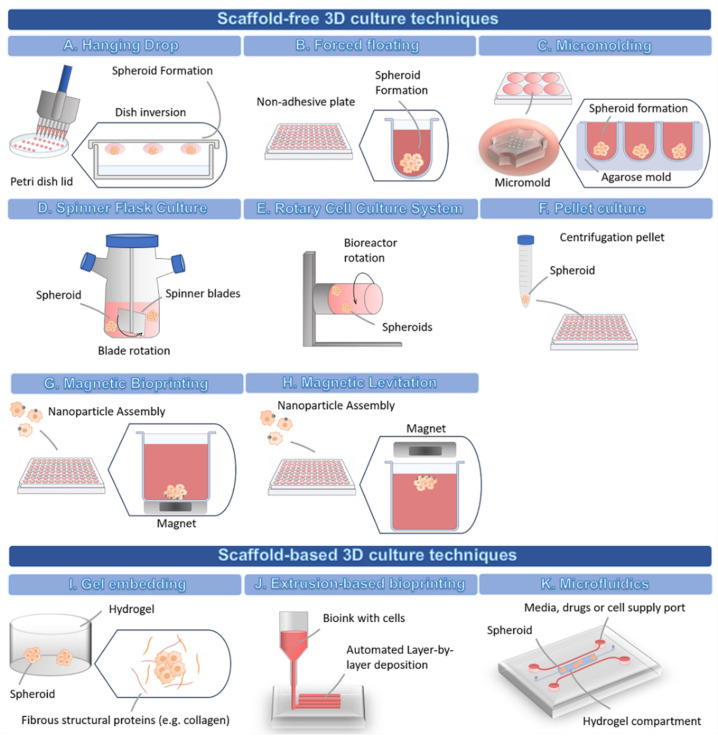
Schematic representation of the different 3D cell culture techniques.

In the hanging drop method (Figure 2A), the cells form a single spheroid by accumulating at the free liquid-air interface formed by their suspension due to the inversion of the dish. Then, the spheroids must be transferred to other standard plates in order to perform cell-based assays, which might reduce the throughput potential of this technique [8]. Many different cell types can originate spheroids through this scaffold-free technique [10].

The forced floating method (Figure 2B) can be carried out using uncoated polystyrene plates or plates coated with a hydrophilic polymer that suppresses cell-substrate interactions, e.g., ultra-low attachment (ULA) plates [8]. Particularly, U-bottomed ULA plates are becoming increasingly popular for their easy use and their compatibility, not only with the majority of drug screening readouts, but also with high-throughput screening, high content analysis and automation systems [8,57,72]. Additionally, the U-shape of the well also allows a single spheroid to be formed at the bottom [57].

In the micromolding technique (Figure 2C), the cells are seeded and allowed to self-aggregate into non-adhesive micro-molds [73]. Agarose is the most used mold material due to its biocompatibility, low toxicity to cells, permeability, and non-adhesive properties [59,73]. The agarose is poured into polymer micro-molds to create smooth non-adhesive agarose molds containing microwells [73]. The solidified agarose molds can then be transferred to 6-, 12-, and 24-well plates and cells are seeded into the microwells to form the spheroids [73]. The polymer micro-molds are autoclavable, possible to reuse many times, and are flexible, allowing an easy detachment from the agarose wells [73]. Additionally, the spheroids can be harvested without using enzymes [59]. The 3D printers can be used to create customized molds with a variety of designs and dimensions [59,73]. As an alternative, commercial molds, such as MicroTissues^®^ 3D Petri Dish^®^ micro-molds, are available for spheroid formation.

The agitation-based techniques, namely the Spinner Flask Culture (Figure 2D), use stirred tank bioreactors that allow cells to spontaneously aggregate into spheroids through continuous stirring conditions, which impair cell adhesion to the surfaces [35]. In stirred tank bioreactors, cells are cultured in high-speed stirring conditions. Thus, this technique is only suitable for cell lines that can withstand high shear stress [74]. The fluid movement allows the establishment of a controlled environment for the renewal of nutrients and transportation of waste away from the spheroid’s surface [74]. The advantages of this technique include the production of high numbers of spheroids, easy exchange of culture medium, and the possibility to cause modifications to the cell culture conditions in situ [29].

The Rotary Cell Culture System (Figure 2E) is another agitation-based technique used to obtain a higher number of large spheroids with a small number of starting cells, thus allowing the setting up of multiple-well plates for drug screening assays [32]. This method has been used to create 3D culture from several cell lines, and also primary cells from glioblastoma, ovarian carcinoma, and melanoma [32], and enables co-culture of multiple cell types [74]. This system possesses lower fluid turbulence, milder shear stress conditions, and higher mass transfer, when compared to other agitation-based techniques [74]. However, a major disadvantage is the fact that this system might originate spheroids displaying differences in morphology, sizes, and density, which may have an impact on the reproducibility and thus lead to variable drug responses [58]. Moreover, this system requires expensive special equipment, which makes this methodology less accessible [74].

Regarding the pellet culture method (Figure 2F), this technique allows the modulation of the spheroid dimension by varying the number of starting cells in the cellular suspension [32]. This method has been used to create spheroids with large diameters and compact aggregates within 24 h following initial centrifugation. However, some studies have reported a lack of compatibility with high-throughput screenings due to the high number of centrifugations required to obtain a proper number of spheroids for testing (each centrifugated vial originates a single spheroid) [32].

Magnetic bioprinting (Figure 2G) and magnetic levitation (Figure 2H) are two techniques with similar principles both employing a nanoparticle assembly technique, where cells treated with nanoparticles aggregate into spheroids or organoids under magnetic forces within a few hours after a magnet is placed on top of the lid (magnetic levitation), or underneath the plate (magnetic bioprinting) [28,61,62,63,75]. In magnetic bioprinting, each well faces an individual magnet, allowing the aggregation of cells in the center of the well through magnetic forces [61,62,63,75]. The main challenge of the magnetic bioprinting approach lies with the need to fabricate magnetic drives with enough precision to allow the proper alignment of the plate wells with the magnets, which is essential for the correct development of spheroids [61].

The main advantages of the scaffold-free techniques are that they are generally simpler and less expensive than the other 3D techniques [76]. Also importantly, scaffold-free techniques allow to co-culture cells in ratios between 1:1 (e.g., more representative of in vivo immune cells infiltration into the TME) and 1:10 [76,77]. In general, scaffold-free techniques can be adapted to automatized pipetting systems and can be applied in high-throughput screens (HTS) [10,78]. For instance, Madoux and colleagues evaluated the effect of around 3300 approved drugs on spheroid cultures grown on 1536-well round-bottom ULA plates [79]. Through a luminescence-based cytotoxicity screen, the authors concluded that the results were significantly different from screens performed on monolayers (76% of the compounds were more cytotoxic in the 2D models). Moreover, the authors were able to assay an average of 10 plates per hour (around 15,000 wells), demonstrating the possibility of using the method to evaluate large compound libraries in an ultra-HTS [79]. Similarly, a study demonstrated the possibility for full robotic automation of magnetic bioprinting for HTS on 1536-well plates [61]. Through the magnetic bioprinting methodology, these authors were able to screen on average 15,200 compounds per day [61].

Unfortunately, regarding the scaffold-free techniques readout, it is particularly difficult to visualize spheroids on round-bottom plates and on hanging drops [75]. Additionally, these techniques might be inadequate for some cell types, which are challenging to spontaneously form spheroids, since different cell lines have distinct adhesion properties [31,75,76]. For this reason, the most suitable spheroid formation method must be established for each cell line. For instance, while the breast MCF-7 and pancreatic BxPC-3 cancer cells can form spheroids spontaneously, the breast MDA-MB-231 and SKBr-3, or the pancreatic Panc-1 and MiaPaCa cancer cells require the addition of a reconstituted basement membrane [31]. Similarly, the prostate PC3 cancer cell line can form loose aggregates, requiring additional ECM components to form spheroids, contrary to the prostate RWPE-1 cancer cell line, which progresses from monolayers to spheroids just by adding 10% FBS [57].

Interestingly, Selby and colleagues published a comprehensive list of the 60 cell lines present in the National Cancer Institute (NCI) that could be used for generating 3D spheroids with diameters ranging between 300 and 500 µm, using ULA plates, providing also information of the optimal cell densities and conditions for these assays [55].

A study from Zanoni and co-workers compared 4 different scaffold-free techniques, namely magnetic levitation, hanging drop, pellet cultures, and the Rotary Cell Culture System, for the formation of spheroids from the human non-small cell lung cancer (NSCLC) cell line A549 [32]. The authors reported that all tested protocols induced the formation of spheroids with variable yield, dimensions, and shapes (spherical, ellipsoidal, 8-shaped, and irregular), which are parameters that highly affect the cell viability and thus, the reproducibility of the experiments. For instance, the magnetic levitation and the hanging drop technique led to the formation of spheroids ranging between 200 and 500 μm in diameter in 7 days, which highly contrasts with the mean diameter of the spheroids obtained by the pellet cultures and rotating wall vessels (around 890 μm in 1 and 15 days, respectively). Thus, in order to reduce the influence of this variability on drug testing, and increase the reproducibility of the assays, these authors proposed to monitor different morphological parameters (diameter, sphericity, area, and volume, among others) by using the open-source software AnaSP. This software provides a quantitative analytical method capable of calculating the effect of these variations, allowing the reduction of bias to a minimum [80,81]. In addition, the techniques evaluated by the authors also vary in terms of the required initial number of cells to obtain sufficient spheroids to fill a 96-well plate: around 0.55 × 10^6^ of total cells for the magnetic levitation and hanging drop, and 30 × 10^6^ cells for pellet culture and rotating wall vessels spheroids [32]. This type of information, on the ability to generate 3D cell culture models with a small number of starting cells, is particularly advantageous when using cell lines with high mortality rates or rare patient-derived cells [33].

#### 3.2.2. 3D Scaffold-Based Culture Techniques

The cells composing a tissue or a tumor reside within the ECM, a non-cellular component, consisting of a complex 3D meshwork of fibrous structural proteins (collagen, elastin, and fibrillin) surrounded by a hydrated gel-like material of glycosaminoglycans, proteoglycans, and glycoproteins [11]. Importantly, the ECM has tissue- and organ-specific biophysical, mechanical, and biological properties, providing not only structural support, but also biochemical and biophysical signals to the cells, which are essential for tissue morphogenesis and function [11]. The ECM composition varies with the type of tissue and the phase of the disease [29]. For instance, alterations in the composition and structure of the ECM occur due to cancer cell- and stromal cell-mediated ECM deposition or degradation, mainly through matrix metalloproteinases enzymatic digestion [29]. The deposition of collagen and hyaluronan consequently impacts the matrix stiffening, the tumor’s metastatic potential, angiogenesis, and drug resistance [29].

Scaffold-based techniques involve the seeding or embedding of cells into natural or artificial matrices (Figure 2I) that can be rapidly crosslinked via physical and/or chemical reactions [8]. Such matrices have been used as an attempt to fully mimic the ECM of the in vivo microenvironment [29], providing cells with a biomimetic niche that instructs cell fate in a similar way to the native ECM, thus allowing the recapitulation of complex processes, such as cell invasion and migration. In scaffold-based techniques, the materials used to form the matrices can be derived from different sources, such as decellularized natural ECM (e.g., Matrigel^TM^, collagen, fibrin, gelatin), natural materials (e.g., alginate, chitosan, dextran, hyaluronic acid), and synthetic materials (e.g., Polyethylene glycol (PEG), Polyvinyl alcohol (PVA), Poly-ε-caprolactone (PCL), and poly(hydroxyethyl methacrylate) (polyHEMA)) [11,29].

Proteins (e.g., collagen, gelatin) and polysaccharides (e.g., chitosan, alginate, hyaluronic acid) are commonly employed to create 3D hydrogel models loaded with cells due to their intrinsic cell-interactive properties or ability to be biofunctionalized in order to present cells with specific biophysical and biochemical cues. In addition, hydrogels can be formed in the presence of cells through a variety of crosslinking mechanisms, including ionic gelation, photopolymerization, and click reactions [68,82,83,84,85,86,87]. Scaffolds composed of natural materials have the advantage of possessing cell-binding ligands, endogenous chemokines, and growth factors, which enhance cell viability and growth, also being naturally recognized and remodeled by the cells [11]. However, natural materials might impair the quality control, reliability, and reproducibility of the assays, due to batch-to-batch variabilities, the undefined composition of the components, the presence of undesired soluble components, and higher speed of self-degradation [8,28,29].

Synthetic materials, aside from being well-defined in terms of chemical composition, have tunable mechanical properties and can be modified with suitable functional groups to engineer hydrogels with controllable degradation rate via hydrolysis, oxidation, and/or enzymatic degradation mechanisms [85,88,89]. In addition, synthetic materials have been explored to create hydrogel networks that undergo dynamic stiffening, recapitulating this aspect in specific diseases, such as tumor progression and fibrosis [28,29,90]. Nevertheless, the downsides of these materials are the lack of sites for cellular adhesion and the requirement for additional ECM proteins, growth factors, hormones, and other biologically active molecules to truthfully mimic the natural ECM [35]. Consequently, cells cultured in these synthetic platforms present inconsistent tumorigenicity, metastatic potential, and drug-resistance phenotypes, when compared to in vivo tumors [29]. Therefore, to overcome this drawback, hybrid scaffolds consisting of the incorporation of natural bioactive (such as growth factors) and bioadhesive molecules (such as peptides) into synthetic materials have been explored. Despite their potential, the production of these hybrid materials often requires time-consuming and multiple steps, limiting their use in high-throughput screenings [29].

Alternatively, several authors have been using decellularized matrices (dECMs), which are obtained from malignant or healthy tissues, or by in vitro ECM production of regenerated tissues constructed from cultured cells [29,91]. The decellularization process consists of the application of cell removal agents (chemical, biological, or physical) to obtain a matrix with a minimal degree of adverse effects on ECM structure and composition [91,92,93]. A decellularized matrix must have the following criteria: less than 50 ng of double-stranded DNA per mg ECM dry weight, less than 200 base pairs of DNA fragment length, and lack of visible nuclear material evaluated through DAPI or hematoxylin and eosin staining [93]. This process provides native ECM components, however, these matrices lack structural and architectural control [29]. Moreover, although tissue-derived dECMs provide greater similarities with native ECM, they have limited supply sources and their composition is highly heterogeneous among cancer patients, which is a challenge for drug screening in cancer research [91].

The scaffolds can be divided into three types: hydrogel scaffolds, paper-based scaffolds, and fiber-based scaffolds, each of them with their own applications, advantages, and drawbacks. Hydrogels, owing to their high-water content, better mimic the hydrated nature, porosity, and viscoelastic properties of the natural ECM. Hydrogels are composed of one or more hydrophilic polymers, whose polymerization pattern allows cell and molecule movements across the pores [27]. The vast variety of materials (biological-derived and synthetic components) that can be used to create hydrogels allows fine-tuning of certain properties, such as porosity, stiffness, and degradation of the matrix [27]. Notably, hydrogels are widely used in 3D culture methods, such as in 3D bioprinting [67] and microfluidics [27].

Regarding paper-based scaffolds, they rely on commercially available scaffolds made of cellulose, which are less labor-intensive but more rigid than traditional hydrogels, and can be folded into complex geometries providing a porous structure for cell growth [27]. The rigidity and thermal stability of paper-based scaffolds allow for a wide range of surface modifications and the use of sterilization techniques. However, they also present limitations as an in vivo mimic, since cellulose-based materials require the addition of ECM proteins. In addition, the fibers of this scaffold are larger than 1 mm in diameter, making them incomparable to the fibrils present in the body (around 500 nm in size) [27].

The fiber-based scaffolds are similar to hydrogels in terms of variety of materials that can be applied to form the scaffold (biological and synthetic components). In this type of scaffold, the fibers can have sizes ranging from 10 µm to 10 nm and have high biological compatibility, which overcomes some of the problems displayed by paper scaffolds [27]. This type of scaffold is also highly tunable in terms of structural properties and strength and has slower degradation capability (especially when using synthetic fibers), allowing better stability over time and at a wide range of temperatures [27].

The scaffold-based approaches have innumerous advantages, however, they also present their own drawbacks. For instance, their thickness and sometimes low transparency make them incompatible with certain imaging techniques (e.g., high content imaging techniques) [94]. Additionally, their viscosity (especially if using collagen or matrigel) can be a challenge for the automation of liquid handling, contrasting with the liquid handling for suspension media and ULA plates, where automation can be accomplished [94]. However, this issue has recently been addressed by the development of extrusion-bioprinting strategies enabling the automated dispensing of both low and high viscosity polymer solutions with high levels of reproducibility and resolution [95,96]. Moreover, the manipulation and polymerization of some natural materials, such as collagen, gelatin, and matrigel, requires temperature and environment control, as well as fast handling to prevent premature polymerization of the matrix [94]. Furthermore, the need to perform additional steps to separate the cells from the matrix can further complicate the biochemical analysis, and the matrix itself can interfere with the colorimetric measurements and detection of fluorescence signals [10,97]. Some natural-derived materials may also interfere with antibody labeling of protein and with detection of ribonucleic acid (RNA) and DNA, due to the presence of endogenous factors [98]. Importantly, the chemical and physical properties of the matrix may also impair the diffusion of certain compounds, such as drugs, with particular impact for drug screening outcomes [98]. In addition, some drugs might form interactions with the scaffold materials, hereby impairing their uptake by cells [35].

Amongst the scaffold-based methods available, the extrusion-based 3D bioprinting (Figure 2J) allows the layer-by-layer deposition of cells, biomaterials, and biochemical factors into 3D constructs with predesigned features, therefore allowing the creation of geometrically complex scaffolds and biomimetic tissue models [34]. This approach also provides the opportunity to control the spatial location of multiple cells, biomaterials, and bioactive factors in 3D, improving the level of biomimicry and, therefore, contributing to enhancing the reproducibility, standardization, and accuracy of the assays [34]. Extrusion bioprinting has also shown compatibility with high-throughput approaches [99] and with cell co-culture approaches [100,101], also enabling the creation of standardized models for the screening of anti-cancer drugs [102,103].

Microfluidics devices (Figure 2K) have also been explored to create 3D models for drug screening. They usually comprise a chip composed of microchambers and microchannels (typically containing a hydrogel compartment), where cells can be suspended in a medium that circulates and accumulates in chambers forming spheroids, or the cells can be embedded within a biomaterial [35]. These devices have several variants and can be applied to encapsulate tumor cells and supporting cell types within hydrogels [6]. For example, it is possible to generate a simple single-channel device with two adjacent hydrogel compartments containing different cell populations [104]. The different sized inlet and outlet ports allow passive pumping of the medium through the gel, without the need for external pumps [104]. This device is compatible with several biomaterials and can be adjusted to the type of question to be answered [104]. There are different models, including more complex microfluidic platforms, with multiple compartments and channels, allowing supplementation with medium, drugs, and other cells (e.g., endothelial cells migrating into the center of the hydrogel under certain culture conditions), as well as the possibility to establish drug and growth factor gradients across the hydrogel [6]. Interestingly, microfluidic systems have been used to model metastatic tumors and to study the effect of drugs on the inhibition of tumor cell migration [105]. All three types of scaffolds (hydrogels, paper-based, and fiber-based) have been integrated into these microfluidic systems [27]. Alternatively, microfluidic devices can be used scaffold-free, by coating the surfaces with specific proteins or polymers, such as bovine serum albumin or polyHEMA, to make surfaces resistant to cell adhesion [64,65,66].

### 3.3. 3D Cell Culture Assay Readouts

One of the main problems behind the transition from 2D to 3D cell culture for drug screening is the fact that tools and equipment available to evaluate cytotoxicity, investigate gene and protein expressions, among other assay endpoints, are difficult to apply to 3D models [10,32]. Nonetheless, many basic and complex techniques have been implemented to analyze 3D models [106].

As reviewed by Brooks et al., drug-response assays aim to evaluate the efficacy of a drug over a range of concentrations. Several drug response metrics can be used, whenever possible, to account for possible experimental variation, initial populations, and number of cell divisions during the assay: E_max_ (the drug’s maximum effect), EC_50_ (drug concentration which achieves half of E_max_), IC_50_ (the inhibition concentration where the response is reduced by half), GI_50_ (concentration that reduces total cell growth by 50%), GR_50_ (concentration that inhibits cell growth rate by 50%), and AUC (the area under the dose-response curve, representing the cumulative effect of the drug) [71]. The GI_50_ and GR_50_ are particularly interesting pharmacology metrics for taking into account the variations in growth rates between different cell lines, a feature that has increasing relevance when comparing 2D (with faster growth) to 3D cell cultures (with slower growth). These two metrics take into consideration the initial population and thus require the inclusion of an additional plate in the assay, in order to measure the initial cell counts prior to drug exposure. Unfortunately, the GR_50_ is not always applicable to 3D systems, since it requires exponential growth of cells throughout the assay, which is a characteristic that is rarely seen in 3D systems when using patient-derived primary cells [71].

In the following section, we present some of the endpoints that can be evaluated on 3D models, as well as the available assays to assess such endpoints.

#### 3.3.1. Spheroid Viability and Cytotoxicity

Facing the lack of standard methodologies for evaluation of cell viability on 3D models, many metabolic assays were developed specifically for this end, including the Perfecta3D-Cell Viability assay and the CellTiter-Glo^®^ 3D Cell Viability assay [32]. These chemiluminescent assays involve the quantification of a luminescent signal that results from the conversion of luciferin to luciferase, as a consequence of the cytoplasmatic adenosine triphosphate (ATP) concentration [56]. Through optimization of the detergent composition and the lysis conditions (such as time of the lysis), it was possible to develop assays that overcome the common challenges associated with 3D cell culture: decreased penetration of dyes/reagents, decreased lytic activity due to the presence of 3D matrices, and the tight cell-cell junctions of the 3D cellular aggregates [107,108]. These chemiluminescent cytoplasmatic ATP detection assays are sensitive, applicable to high-throughput screens, and allow a simple workflow and data analysis [32]. Moreover, the existence of ready-to-use kits enhances the standardization of the assays and reduces time consumption, as they combine the lysis with luminescent signal generation into one step [32].

The standard colorimetric methods, such as the acid phosphatase activity assay (based on dephosphorylation of *p*-nitrophenyl phosphate) [109], the Alamar blue assay (based on resazurin reduction) [110], the MTT assay (based on tetrazolium reduction) [111], the Trypan Blue exclusion assay (based on its exclusion by live cells’ intact membranes) [32,56,112], and the lactate dehydrogenase (LDH) activity assay (based on enzymatic conversion of tetrazolium into a red formazan product) [111,113], are still used in 3D models. Nevertheless, these assays were found not to be efficient in 3D spheroids and matrices, usually due to incomplete probe penetration and limited sensitivity [6,57,108,111].

Interestingly, Zanoni and co-workers compared three viability assays on large spheroids following a 72-h exposure to albumin-fenretinide nanocapsules (4-hydroxy(phenyl)retinamide). These authors found a dose-related efficacy of the drug on both Perfecta3D-Cell Viability assay and CellTiter-Glo^®^ 3D Cell Viability assay, with similar degrees of data variability [32]. The trypan blue exclusion test, however, showed high cytotoxicity from the lowest drug concentration and a high level of data variability, and no dose-dependent effect of the drug was observed [32]. In the end, after pairing the test viability results with a light-sheet fluorescence microscope analysis, the authors concluded that the CellTiter-Glo^®^3D Cell Viability assay provided a more accurate evaluation of the viability of spheroids up to 650 μm in diameter [32].

Furthermore, Ho and colleagues employed the MTT assay to perform a high throughput screening on MCF-7 spheroids. The authors also compared the obtained results with the ones obtained with the LDH release assay, concluding that the MTT was a better indicator of cytotoxicity. Indeed, the LDH could not detect a wide range of cytotoxicity due to high basal background reading, which was attributed to the presence of cell death and necrosis in the central region of the spheroid [111]. These results suggested that large spheroids produce apoptotic signals that can sometimes be even greater than the ones induced by drug treatment, thus interfering with the cytotoxicity measurements [33,57]. Under these circumstances, analyzing small spheroids may be preferable [33], or coupling the cytotoxicity assay with other measurements, such as fluorescent markers of apoptosis [57].

For instance, another study demonstrated the need for both visual and quantitative assessment of drug-mediated effects on spheroid cell viability and morphology [31]. The breast cancer MDA-MB-231 and MCF-7 spheroids, after being exposed to a selected chemotherapeutic cocktail or tamoxifen, respectively, displayed variations in morphology (verified by light microscopy): MDA-MB-231 spheroids suffered an increase in compactness, whereas MCF-7 spheroids became frayed and uneven. However, the CellTiter-Glo^®^3D Cell Viability assay confirmed the decrease in viability of both spheroids after their respective treatment, proving that the treatment effects on morphology parameters can be cell- and treatment-specific [31].

The evaluation of the cytotoxicity in 3D co-cultures through these common viability tests still leads to potentially confounding results, since it is not possible to isolate the response of cancer cells within the mixed culture [71]. For instance, the presence of stromal cells could increase the survival of cancer cells after drug exposure [114], compromising the calculation of IC_50_ or EC_50_ values [71]. Under such circumstances, it is more appropriate to use cells that express a reporter transgene or perform image-based assays (e.g., using confocal or multiphoton microscopy) or flow cytometry, allowing the multiplexing of different fluorophores to discriminate cell types and mark cell viability [39,71,115].

Label-dependent methods for viability assessment, such as fluorescence imaging and luminescence assays, lack sensitivity when analyzing large spheroid cultures with hypoxic and acidic compartments, due to the low penetration depth [116]. Moreover, these techniques, in general, do not allow real-time monitoring of drug response [117]. Consequently, label-free approaches are being developed, for a more robust viability readout on 3D cell culture models.

Optical coherence tomography (OCT) is an optical imaging technique that allows non-invasive longitudinal visualization of live cells with subcellular resolution at depths exceeding several millimeters [116]. OCT can be used as a quantitative method to assess treatment response which, given its fast scanning speed, allows a high-throughput structural imaging of 3D cell culture models [116]. Jung et al. compared the commonly used LIVE/DEAD Viability/Cytotoxicity Assay with the OCT and reported that the latter was able to surpass the limitations of the LIVE/DEAD assay, by providing metrics on treatment response with higher sensitivity than the LIVE/DEAD assay [116].

Convolutional Neural Network Image Analysis is another label-free method based on bright-field imaging and image processing for the estimation of spheroid viability and IC_50_ of chemotherapy drugs [118]. Some advantages of this method over others, such as fluorescence-based methods (e.g., LIVE/DEAD assay), are the non-invasive and non-destructive manner of providing a viability estimation, the possibility of reducing the processing time through machine learning, the low cost of the technique, and the absence of other disadvantages associated with fluorescence-staining (such as fluorescence imaging time) [118].

Another label-free alternative method for real-time spheroid imaging and viability monitoring is electrical impedance tomography [117]. This method employs a miniature sensor for the measurement of the electrical properties of cells to determine cell concentration, size, viability, proliferation, drug response, and other cell activities and characteristics [117]. The conductivity images are then reconstructed with a speed below 0.3 s and if more than one pair of electrodes is used, it is possible to investigate the spatial distribution of the conductivity [117]. However, this technique has a low detection limit since it is only possible to obtain a good correlation between the reconstructed conductivity variation and the cell mortality rate when the latter is at 20% or higher [117].

#### 3.3.2. Microscopy Techniques

Many microscopy techniques have been employed to study 3D tumor models. Imaging techniques are particularly advantageous over plate reader-based luminescence or fluorescence, as they do not require spheroids disruption and allow multiple readouts [56]. Size and shape are the primary parameters evaluated by microscopy techniques for real-time monitoring of the drug’s effect on 3D cell cultures [57]. Brightfield microscopy has been used to observe the damage caused by drug exposure on the architectural structure (size and shape) of spheroids over time and to monitor complex tumor processes, such as invasion and angiogenesis [32,57,119]. Through transmitted light imaging, it is possible to visualize spheroid size and to some degree also density [56]. Alternatively, scanning electron microscopy and transmission electron microscopy have been used to assess the morphological (either at the surface or at the lumen side) and ultrastructural features of 3D cell culture models [120,121,122]. In addition, they have also been used to characterize the permeability, uptake pathways, and intracellular fate of nanoparticles in spheroids [121,123]. However, these techniques have endpoint destructive assays and thus cannot be used for real time monitoring of 3D cell culture models [117]. Fluorescence microscopic techniques add more information to the assays, as fluorescent markers can be used to evaluate viability, DNA presence, and apoptosis, among other parameters [56], allowing to infer the drug’s mechanism of action [57].

Importantly, 3D model imaging is affected by several issues, namely poor light penetration, light scattering by cells, and high background due to out-of-plane fluorescence [56]. In contrast to 2D cell culture, where imaging techniques involve the capture of a single xy image, 3D models can be more informative when recording a series of xy images captured at fixed steps across a z axis, forming a z-stack [98]. The z-stacks are used to get images with varying depths of the spheroid, preferably with a collection of 11–18 stacks per spheroid and leaving 18–35 μm of distance between each slice [31]. Z-stacks can then be processed using open-source software, such as ImageJ (using the z-projection function), which can apply a maximal projection algorithm to combine maximum intensity pixels from z-stacks into a final image, giving an overview of the staining throughout the 3D model [31,56]. Although increasing the number of z images captured can result in a higher number of counted objects in the maximal projection image, a practical compromise between better sampling of cells and increased image acquisition time might be necessary [56]. Depending on the size, it can also be possible to capture spheroids with objective magnification as low as 4× [98]. In order to acquire one field of view per well, however, care should be taken to ensure that the 3D model’s size is within the instrument imaging capacity. For instance, on CellInsight NXT, the diameter should be below 800 μm or even 600 μm, if the shape is irregular or if the 3D model is not centered in the well [57]. Nonetheless, higher magnification objectives provide more information on the 3D model structure and subcellular content (e.g., through single-cell resolution) [56]. However, a disadvantage of the use of a higher magnification is the increase in the image capture time due to the need to multiply the number of xy fields and z planes to capture the same number of objects [98]. Consequently, image capture at higher-magnifications impacts the suitability of this technique for high-throughput approaches [98].

Regarding microscopy, the confocal microscopy may be preferable to acquire detailed information on the subcellular morphology and molecular distributions, and can provide less background and sharper images, although the compatibility with high-throughput screening is limited [6,10,56]. The confocal microscopy can also be used to analyze cancer stem cells by labeling the cells with the nucleoside analog 5-ethynyl-2′-deoxyuridine (EdU) and nuclear stain (TO-PO-3), offering a non-destructive method for the quantification of EdU label-retaining cells in the 3D structure [124].

Concerning the wide-field fluorescence microscopy, the imaging capture time is improved compared with confocal imaging, but it requires post-imaging deconvolution in order to reduce out-of-focus fluorescence signal from nearby cells that interferes with the precision of the measurements [98,115]. Light-sheet fluorescence microscopy (LS-FM) is another advanced method of fluorescence microscopy that was developed for mapping 3D structures in large samples [32,35], with limited out-of-focus signal by exciting fluorescence only in a thin sheet [115]. Particularly, the single plane illumination microscopy (SPIM) allows no photodamage caused either above or below the plane of focus, as it only excites the fluorophores present in the light sheet (illumination is perpendicular to the axis of the microscope objective), and is compatible with longer imaging time and long-working-distance lenses [10,28]. By allowing sequential focal sectioning of the sample, SPIM enables the capture of high-resolution images [28].

Spheroids can also be stained with fluorescent probes on microscopy techniques, which have the advantages of not requiring spheroid fixation or dissociation, being applicable to different types of cells [57]. However, staining time will need to be optimized depending on dye structure/properties, as well as integrity and mass of the spheroids, since staining of spheroids differs from the staining of cells in monocultures due to reduced or uneven rate of dye penetration into the spheroid mass [57]. Many fluorescent probes have been used on spheroids grown in various 3D cell culture methods that aim for: nuclei staining (mostly Hoechst 33342, but also DRAQ5 and SYTO11); indication of viability and/or cytotoxicity (mostly Calcein-AM/Ethidium dimer, but also Propidium iodide, SYTOX Green, CellTox Green and MitoTrackers, among others); indication of reactive oxygen species and superoxide (Dihydroethidium, DCFDA and MitoSOX Red); measure of glutathione (Chlorobimane), hypoxia, and O_2_ gradient (HypoxiSense 680, LOX-1, Cyto-ID^®^ HRDR, NP NanO_2_, among others); mitochondrial activity (TMRE, TMRM); proteolysis (ProSense^®^ 680); cell labeling and tracking (CellTrackers and Vybrant DiD); pH gradient (BCECF); and apoptosis (CaspGLOW Red, Nucview 488, CellEvent™ Caspase-3/7 Green, LysoTrackers) [31,56,57,72]. While using many probes increases the information that can be gathered, care should be taken in order to not interfere with spheroid growth nor with the drug effect, nor affect the fluorescence signal detection when using more than one probe together [57].

Live cell imaging of spheroids can be achieved through time-lapse microscopy. This technique has been used in several studies such as: (i) to visualize stem cell activity in pancreatic cancer expansion by direct genetic lineage tracing with a dual-recombinase system [125]; (ii) to assess the role of cancer-associated fibroblasts (CAFs) in the migration of epithelial tumor cells [126] and endometrial tumor cells [127]; and (iii) to monitor invasion and metastasis of fluorescently labeled cancer cells [128]. Image processing and data quantification can then be accomplished by using a Fiji Software macro [128]. Ahmed-Cox and colleagues developed an analysis platform termed the “Determination of Nanoparticle Uptake in Tumor Spheroids” (DONUTS) for quantitative and automated analysis of confocal time-course data of nanoparticles in live tumor spheroids, thus overcoming the challenge of static approaches for measuring nanoparticle accumulation in spheroids [129].

Importantly, as previously discussed, imaging 3D structures is particularly challenging, especially when they float freely in the media without adhesion to any substrate. The use of inert and optically clear cell mountant (such as CyGEL™), with refractive indexes similar to water, allows the immobilization of spheroids while maintaining their viability and morphology, thus enabling the acquisition of high-quality images of live cells [124]. In addition, 3D structures can be fixed, paraffin-embedded, sectioned, mounted on slides, and stained for immunohistochemistry [122,130].

#### 3.3.3. Other Single-Endpoint Analysis

Flow cytometry has also been used to analyze several parameters on 3D structures, such as apoptosis, cell viability, proliferation kinetics, and cancer stem cell phenotype [28]. For instance, apoptosis has also been evaluated on spheroids using flow cytometry (staining with Annexin V-FITC and propidium iodide solution) [111]. Flow cytometry analysis of CD44^+^/CD24^−/low^ and CD133 is also performed to investigate cancer stem cells (CSC) niches in spheroids [124]. The downside of flow cytometry in spheroid analysis is that the information regarding the spatial distribution of the marked cells is lost due to the complete dissociation of the spheroids (through an enzymatic reaction), which destroys spheroid integrity and affects cell viability [28]. However, the COPAS BioSorter, an improvement on flow cytometry technology, can be used for particles ranging between 20 to 1500 μm, allowing analysis of intact spheroids [28].

Western blotting is also available to analyze protein lysates from 3D cell cultures [31]. However, immunoblotting of 3D cell culture proteins, similar to flow cytometry analysis, inevitably leads to the loss of important information on the spatial arrangement [31,131]. Another available detection method that can be used is the immunohistochemistry analysis, which evaluates specific protein expression and phosphorylation without affecting the spatial arrangement of the 3D cell culture model [31,131]. Many other detection methods are possible to be performed using 3D cell cultures, such as the enzyme-linked immunosorbent assays (ELISA), RNA analysis, quantitative PCR analysis, and matrix-assisted laser desorption/ionization (MALDI) imaging mass spectrometry (e.g., to examine the localization of proteins, small molecules, as well as drug and active metabolites in the 3D models [132]) [27].

For example, the metabolomic analysis of 3D cell cultures was also performed, using a stacked-paper TRACER platform [133]. In this technique, the cells were encapsulated in a collagen hydrogel on a paper-based strip, rolled around a central oxygen impermeable mandrel, to form a stacked configuration [133]. The structure is quickly disassembled by unrolling, and the location on the strip can be mapped to the location within the 3D structure, allowing the assessment of spatial variations of the metabolite profile. By coupling this method with liquid chromatography-mass spectrometry, the authors identified 23 metabolites whose profiles varied across the oxygen gradient. These metabolites were associated with glucose metabolism and mitochondrial function, and evidenced an increase in glycolysis, deregulation of tricarboxylic acid (TCA) cycle, and increased fatty acid oxidation in the innermost hypoxic layers [133]. However, one of the limitations of this platform is the impossibility to distinguish between the intracellular and extracellular metabolites, thus impairing the full analysis of the metabolic pathways [6,133].

#### 3.3.4. Multiparametric Analysis and High-Content Imaging

High-throughput screens usually evaluate a single parameter, such as cell viability, proliferation, or single genes to evaluate spheroids’ treatment responses [98]. Nonetheless, although analyzing a single endpoint may be advantageous for readout interpretation, we might be overlooking a lot of information that could have been extracted from the model [98]. Thus, multiparametric analysis and high-content phenotypic screening techniques are arising to assess complex cellular response patterns facing drug exposure [28,98,134].

High-content screening techniques are advantageous since they do not require dissociation of the spheroid structure, and allow simultaneous quantitative analysis of multiple parameters in high-throughput screens: total cell count, density, dimensions, growth kinetics, nuclear mass, among others [28]. This type of analysis has also been successfully performed in a semi-automated manner on several matrix-embedded 3D culture models [57,112,135,136].

For instance, Sirenko and co-workers developed a high-magnification high-content imaging and image analysis methodology to perform a multiparametric characterization of spheroid phenotypes, as well as to determine IC_50_ values using different output parameters [56]. The authors exposed drug-treated spheroids to several fluorescent dyes: Calcein AM (measures metabolically active cells and viability), Hoechst (measures total cell count and nuclear shape), and Ethidium Homodimer-1 (an indicator of dead or necrotic cells due to damaged membrane penetration), and combined Z-stack images into a single maximal projection image to enable efficient segmentation and counting of nuclei. The multiparametric image analysis, performed on MetaXpress CME, allowed quantification of several biological outputs, such as the number of live, dead, and total cells, spheroid diameter and area, and calcein-AM intensity [56]. This method could be extended to other cell lines or combinations of multiple cell types, with additional fluorescent markers for readouts related to hypoxia, mitochondria, cytoskeleton, and kinase activation [56].

In another study, the effect of several anti-cancer drugs (cisplatin, docetaxel, etoposide, ARN-509 and the preclinical anti-cancer drug MLN4924) was described through the determination of the EC_50_ values, using high-content analysis of spheroid assays [57]. VCap and LNCaP prostate cancer spheroids grown in U-bottomed 96-well microtiter plates were scanned once a day on the CellInsight NXT High Content Screening Platform, and the images were analyzed using HCS Studio software. It was possible to evaluate several parameters of the spheroids, such as area, shape, and apoptosis through CellEvent and LysoTracker Deep Red fluorescent markers [57].

Several open-source and commercial softwares are able to process 3D images, although not all can analyze high-throughput and high-content image datasets in an automated manner [98]. In 2016, Li and co-workers provided a list of high-throughput imaging systems, including hardwares and softwares, and concluded, to their knowledge, that the Harmony software (designed for PerkinElmer HCS instruments) was the only high-throughput imaging platform capable of true 3D high-content analysis [137].

## 4. Drug Screening Using 3D Models

### 4.1. 2D vs. 3D Models: Disparity in Testing Outcomes

Many studies have demonstrated that responses to treatment in 3D cell culture models are more similar to the observed in vivo, when compared to those of 2D culture, emphasizing the use of human 3D cultures to study, with more accuracy, the efficacy of different anti-cancer treatments [92,138,139]. Indeed, it is common to observe different outcomes between 2D and 3D models, when treating the same set of cell lines with the same compounds. For instance, Amaral et al. employed two different 3D cell culture techniques, the hanging drop and the forced floating with ULA plates, to form human bladder cancer RT4 spheroids [8]. The spheroids formed with either of these techniques have reduced cell growth, reduced metabolism, and had higher resistance to doxorubicin, when compared to the cells grown in 2D cultures [8].

Another study demonstrated that bioprinting of cervical tumor Hela cells using hydrogels composed of gelatin, alginate, and fibrinogen originated spheroids with higher proliferation rate, upregulation of ECM-related proteins, such as matrix metalloproteinase (MMP), and increased resistance to paclitaxel compared to Hela cells grown in 2D cultures [68]. Similarly, 3D bioprinted glioma (U87) and glioma stem (SU3) cell models, using gelatin, alginate, and fibrinogen hydrogels were reported to be more resistant to temozolomide, than the 2D monolayer models [67], emphasizing the influence of the 3D cell model on drug sensitivity.

Karlsson et al. evaluated the 72-h exposure of colon cancer HCT-116 spheroids (made within 3 or 6 days), to melphalan, 5-fluorouracil, oxaliplatin, irinotecan, and the experimental drugs acriflavine and VLX50 (CD02750), using a 96-well NanoCulture^®^ plate (SCIVAX USA, Inc.,Woburn, MA, USA). The authors showed that spheroids contrast from HCT-116 cells cultured as monolayers, in terms of expression of cell adhesion molecules (E-cadherin and laminin), proliferation markers (Ki67), anti-apoptotic proteins (p21), stem cell markers (CD44), hypoxia-associated genes, and cell cycle and DNA replication genes. Regarding drug sensitivity, whereas monolayer cells were sensitive to all the tested drugs, the 3D cultures presented lower sensitivity [7].

Interestingly, the ability of drugs to kill resistant cells selectively over their drug-sensitive parental cells, a phenomenon known as collateral sensitivity, has also been reported in 3D cell cultures. Although spheroids and tumor stem cells normally display higher resistance to drugs, when compared to cells grown as monolayers [140], breast cancer stem cells cultured as mammospheres into polyHEMA-treated culture flasks have been reported to exhibit collateral sensitivity towards Cajanin stilbene acid derivatives [141]. Additionally, the authors reported a higher sensitivity of MCF-7 monolayer cells over the MCF-7 mammospheres to clinically established drugs, such as 5-fluorouracil and docetaxel, as well as to natural products’ derivatives, such as artesunate and shikonin [141].

Although 3D models typically display increased resistance to drugs when compared to 2D cell cultures, especially due to insufficient penetration of the drugs into the core of the 3D structures, some might actually be more sensitive due to the specific drugs’ mechanisms of action [56]. For example, several cancer cell lines, such as MDA-MB-231, U-87 MG, KNS42, and LICR-LON-HN4 were treated with 17-AAG (a HSP90 chaperone inhibitor), PI-103 (a PI3 kinase/mTOR inhibitor), or CCT130234 (a PLCγ inhibitor) in 2D and 3D cultures [119]. The results demonstrated that those cells in 3D cultures exhibited increased resistance to almost all drugs, with the exception of the PI3 kinase inhibitor [119].

Furthermore, drugs that are more effective in highly proliferative cells and depend on the interaction with the DNA during cellular replication (e.g., carboplatin, cisplatin, doxorubicin, oxaliplatin, methotrexate, and paclitaxel) might not be as successful in 3D cell culture, since cells in 3D grow slowly and have subpopulations of dormant cells [35,142]. For instance, breast cancer cell lines (BT-549, BT-474, and T-47D) exhibited greater doxorubicin and paclitaxel resistance in 3D culture, associated with decreases in cleaved-PARP and caspase-3 expression, higher hypoxia levels, and fewer Ki-67 positive cells (indicating an increase in cells in the G0 phase), when compared to 2D cultures [142].

### 4.2. The Impact of the TME on Drug Screening Outcomes

3D cell cultures are advantageous for enabling the direct co-culture of several cell types, namely tumor cells and stromal cells, and thus more accurately representing a real tumor. The tumor-stroma is composed of supporting cell types (such as CAFs, immune cells, endothelial cells, mesenchymal stem cells, adipocytes, etc.) and ECM components [143,144,145]. Stromal activation promotes the transition from normal tissue homeostasis to the development of a microenvironment that promotes tumor survival and expansion, drug resistance, immunosurveillance evasion, and angiogenesis [143,144,145]. Stromal cells secrete growth factors, cytokines, and chemokines that stimulate the growth and survival of cancer cells, acting as chemoattractants of other cells into the tumor [105,146,147]. Stromal cells can also modulate the efficacy of the therapy by influencing drug access to the tumor, or by protecting the tumor cells from the effects of the drugs [146].

Cancer cells represent a small subset of the tumor’s composition, as the main contributor to the tumor’s mass is the ECM, which is secreted by cancer cells but predominantly by fibroblasts, being responsible for the tumor’s stiffness and density [148]. The TME is characterized by low extracellular pH and a high level of hypoxia, which modulates dormant phenotypes of tumor cells, and is associated with therapy resistance and poor prognosis [41]. Therefore, it is important to model aspects of the TME, namely the 3D architecture, to recapitulate the gradient of soluble factors, pH and oxygen, the ECM biophysical and biochemical properties, and the interaction of tumor cells with multiple stromal cells, for the effective screening of new drugs with potential anti-tumor activity.

Importantly, ECM physicochemical and mechanical properties, such as stiffness and cell-mediated remodeling, are emerging as important microenvironment factors that influence cellular response to therapy. For instance, through cell encapsulation in alginate hydrogels, Shin and Mooney were able to assess the effect of the ECM stiffness on the chemosensitivity of a panel of acute myeloid leukemia cell lines. The authors reported that the matrix stiffening influences the proliferation of some of the cell lines, while matrix softening confers resistance to several drugs but increases sensitivity to drugs against protein kinase B (PKB or AKT). These results were further confirmed in vivo, using the same hydrogel system in a xenografted mice model [149].

Interestingly, Rijal and Li evaluated the impact of different scaffolds (decellularized mouse breast ECMs, Collagen, Laminin-rich ECM, and PLGA) on the sensitivity of Estrogen-Receptor-positive breast cancer cell lines (T47D and BT474) to tamoxifen (4-hydroxytamoxifen) and paclitaxel [92]. The effect of these drugs on the inhibition of the cell proliferation was less pronounced when cells were grown on decellularized ECMs, when compared to the other scaffolds, demonstrating the importance of the ECM composition in the tumor’s drug sensitivity [92].

The importance of modeling hypoxia for drug screening has also been demonstrated in 3D cell culture [142,150,151]. Hypoxia-inducible factors (HIF) are examples of proteins that are upregulated in spheroids, when compared to cells cultured in 2D, as a result of the hypoxic environment, which contributes to therapy resistance (for example through upregulation of the multidrug resistance gene (MDR1)) [150]. Doublier and co-workers reported an increase in HIF-1 expression and its target genes, together with an upregulation of P-glycoprotein (P-pg) expression and activity, which was consistent with a decrease in doxorubicin accumulation in MCF-7 cells cultured as spheroids, when compared to these cells cultured as monolayers [150]. The authors observed that in the presence of a HIF-1α inhibitor, 3-(5′-hydroxymethyl-2′-furyl)-1-benzylindazole (YC-1), the spheroids displayed a decrease in P-gp expression, which led to increased intracellular accumulation of doxorubicin and consequently of caspase-9 activity [150].

Moreover, U251 glioma and U87 astrocytoma cells, grown as spheroids under hypoxia or normoxia conditions, exhibited increased but distinct resistance to apoptosis (measured by caspase-3 activity) after exposure to doxorubicin and resveratrol [151]. Similarly to the previous study, under hypoxia conditions, the spheroids exhibited higher resistance to apoptosis compared to cells cultured as monolayers, in this case via an increased expression of the anti-apoptotic proteins BCL-2 and survivin [151].

Interestingly, Imamura and co-workers cultured six different breast cancer cell lines in 3D cultures, and reported that only BT-549, BT-474, and T-47D cell lines were able to form dense spheroids, exhibiting hypoxic areas in their structures and greater resistance to paclitaxel and doxorubicin treatment. The other cell lines, in contrast, formed loose spheroids and showed drug sensitivities similar to those found in the 2D cultures, suggesting a role for hypoxia and tumor density in the resistance to therapeutics [142].

Furthermore, the hypoxic environment found in spheroids can be particularly prejudicial for the effectiveness of drugs known to induce DNA and cell membrane damage (e.g., 5-fluorouracil, cisplatin, doxorubicin, and irinotecan), which produce reactive oxygen species as part of their effective anti-cancer effect [35]. Consequently, targeting the dormant and hypoxic cell populations in 3D cell cultures is emerging as a therapeutic strategy to counteract anti-cancer drug resistance. For instance, Wenzel et al. performed a 384-well high content screening in breast, prostate, colorectal, and primary colon cancer spheroids (which contained hypoxic cores), and identified nine compounds that specifically target hypoxic cells through inhibition of the respiratory chain [152]. The authors also noticed that the drug-induced cell death in the core regions was dependent on the extracellular glucose concentration (higher glucose levels translated into lower cell death) [152]. Moreover, the combination of a respiratory chain inhibitor (metformin or antimycin A), to target the dormant hypoxic cells, with a general anti-cancer drug (paclitaxel or cisplatin), which targets proliferative outer cells, resulted in an improvement of the overall drug response [152].

In fact, many authors have reported the relevance of 3D co-cultures in drug screening outcomes. Through 3D bioprinting, Wang et al. demonstrated that adipose-derived mesenchymal stem cells (ADMSC) contributed to 21PT breast cancer cells’ resistance to doxorubicin, even without physical contact between the two cell types in the 3D model [34]. Additionally, they reported a correlation between the increased thickness of the ADMSC layer (a recapitulation of the status of obesity) and the formation of a more hypoxic microenvironment, changes in stiffness, as well as increase of secretomes and resistance to doxorubicin [34]. In addition, Logsdon and co-workers reported that differences in the co-culture ratio of MDA-MB-231, HCC38, and MCF-7 breast cancer cells to fibroblasts originated variations in the chemosensitivity of tumor cells to doxorubicin [153]. In particular, MCF-7 cultured alone showed higher sensitivity to doxorubicin, when compared to the two triple-negative cell lines; however, when in co-culture, the MCF-7 cells showed reduced response to doxorubicin (even at the low ratios), indicating that they were protected by fibroblasts independently of the cellular ratio. Meanwhile, the MDA-MB-231 and HCC38 cell lines, at low ratios of tumor to stromal cells (e.g., 4:1) presented higher resistance to doxorubicin [153]. Similarly, when A375 melanoma cells were co-cultured with HFF1 fibroblast cells (ratio 1:1) in 3D collagen-based matrices, and were exposed to varying doses of dabrafenib and trametinib, the HFF1 exerted a protective effect over the A375 cells [115].

Interestingly, Bai et al. used a microfluidic platform with a collagen scaffold to evaluate the impact of endothelial cells (HUVECs) co-cultured with lung adenocarcinoma cells (A549 cell line) or bladder carcinoma cells (T24 cell line) on tumor cell invasion and chemosensitivity to four drugs (known to interfere with epithelial-mesenchymal transition (EMT) signaling pathways) [154]. The authors reported an increase in cell dispersion, an indicator of EMT progression, as a consequence of the secretion of growth factors (e.g., HGF and FGF-2) by the endothelial cells, which was inhibited by drug treatment in co-cultures with A549 cells, but not in co-cultures with T24 cells [154].

In another study, Hoffmann and colleagues investigated the effect of various drug treatments on colon cancer spheroids (from Caco-2 and DLD-1 cell lines) co-cultured with peripheral blood mononuclear cells (PBMCs) or CAFs of colorectal origin [155]. The authors observed that different microenvironment compositions altered the spheroid response patterns. Interestingly, PBMCs increased resistance of spheroids from both cell types to 5-fluorouracil/oxaliplatin treatment, but decreased the resistance of DLD-1 spheroids to 5-fluorouracil/irinotecan. Regarding CAFs, they decreased resistance to 5-fluorouracil/irinotecan in Caco-1 spheroids, but had no impact on the sensitivity to 5-fluorouracil/oxaliplatin. More importantly, the authors compared the obtained results to the ones obtained when using 16 colon cancer patient tissue-derived 3D models; three distinct response pattern subgroups were revealed in the patient-derived 3D models, which could not be detected in the 3D cell line-derived models, highlighting the superiority of the patient-derived models over cell line-derived spheroid models in resembling the human tumor-stroma signature [155].

Yi et al. combined 3D bioprinting and microfluidics to create a human-glioblastoma-on-a-chip for the identification of patient-specific responses to chemoradiotherapy [103]. These patient-specific ex vivo models, consisting of patient-derived tumor cells, HUVECs, and decellularized extracellular matrix from brain tissue, reproduced the clinically observed patient-specific resistances to concurrent chemoradiation and temozolomide treatment. Moreover, the combination of bioprinting and microfluidics originated glioblastomas-on-a-chip within a reasonable timeframe (1–2 weeks), offering an important advantage in a clinical setting for medical decisions regarding treatment, given the fast progression of the disease and its high lethality [103].

Interestingly, Lee et al. suggested that the use of simpler 3D co-culture assays (e.g., cultures containing only CAFs and tumor spheroids) could be employed when the drug’s target is well-established [148]. Nonetheless, triple cultures involving cancer cell lines, fibroblasts, and immune cells were also established using 3D cell culture techniques [156]. For instance, Howes et al. established a 3D cell culture model with triple cultures (breast cancer BT-474 cells, human fibroblasts, and HUVECs) using 96-well round-bottom ULA plates. The authors identified 12 compounds from the NCI’s Approved Oncology Drug library (mainly targeting receptor tyrosine kinases or microtubules) that exhibited greater selectivity for the triple cultures over the normal co-cultures (fibroblasts with endothelial cells) [131]. Furthermore, they observed differences between the 2D and 3D cultures in terms of the spatial organization, intensity, and protein levels of key signaling molecules, as well as in terms of sensitivity to drugs [131]. Moreover, Kenny et al. developed a multilayered 3D culture model adaptable for HTS assay, consisting of primary human fibroblasts, mesothelial cells, and human ovarian cancer cell lines (HeyA8, SKOV3ip1 and Tyk-nu). Using this model, they identified small molecular inhibitors of cancer cell adhesion, invasion, and early metastasis [157].

In addition to the studies comparing drug response between 2D and 3D cell culture models, in the presence or absence of stromal cells, several studies have indicated the similarity between the drug screening outcomes of 3D cell culture models and in vivo models. Recently, it was reported that 3D patient-derived organoid models of human pancreatic cancer were able to recapitulate the in vivo tumor biology and maintain patient-specific transcriptional profiles and drug response, as well as cancer stem cell functionality and in vivo tumorigenicity [158]. In another study, a 3D ovarian tumor model of clinically relevant size was grown in an alginate hydrogel with the MIVO^®^ fluidic device to resemble the human circulation and drug extravasation reaching the tumor mass, and to compare this model with 3D spheroid models grown under static conditions and with an in vivo xenograft model [159]. The authors exposed these three models to cisplatin at comparable concentrations and reported that, under static conditions, the 3D tumor tissue displayed resistance to the cytotoxic agent over time, while under fluid-dynamic conditions, the cancer cell viability decreased over time. Thus, the results obtained with the fluid-dynamic conditions agreed with the ones observed in vivo and contrasted with the ones obtained with the in vitro static approach [159].

Taken together, these studies highlight key parameters that should be considered when establishing a 3D cell culture model for drug screening purposes: (a) 3D model size, enabling the recapitulation of pH, oxygen, and soluble factor gradients, as well as the 3D tumor architecture (outer proliferating cells, quiescent and hypoxic inner cells, and necrotic cores); (b) direct co-culture of tumor cells with multiple stromal cells, applied in biological and disease-relevant cell ratios; (c) selection of appropriate and relevant ECM compositions, enabling the recapitulation of in vivo tumor physicomechanical properties (e.g., stiffness, degradation, porosity) and biochemical composition (e.g., cell-cell adhesion, growth factor presentation).

## 5. Conclusions

The scientific community is becoming increasingly aware of the limited predictive validity of the current preclinical models for drug development. The prioritization of a higher screening throughput over the development of more representative in vitro models capable of recapitulating disease-relevant aspects of in vivo tumors, explains the observed lack of efficiency in drug development. Thus, 3D models are emerging to bridge in vitro 2D cell models and in vivo models, gaining popularity for their physiological relevance and ability to replicate characteristics associated with intercellular interactions and interactions of tumor cells with the extracellular matrix. 3D models can enhance the predictive power and provide a reduction in both financial and time costs during later stages of the drug development timeline, allowing the early detection of ineffective agents, thus reducing the risk of drug withdrawal from the market.

Unfortunately, the large variabilities between 3D models limit their level of standardization, reproducibility, and their use as preclinical tools for drug development. Ideally, the model must be complex enough to allow the replication of key microenvironmental cues and yet be reproducible, and allow a straightforward interpretation of the results. In our opinion, automated 3D bioprinting might become the technique of election to pair the replication of complex microenvironments with high levels of standardization, reproducibility, and screening throughput. In the future, coupling 3D models with high-throughput screening, high content imaging approaches, and advanced microscopic techniques will allow these models to become fundamental tools in pharmaceutical development and biomedical research. In order to help scientists adapt their work from monolayer to 3D cell cultures, we believe that a systematic assembly of 3D culture methods and endpoints, as described in this review, will be of great benefit.

## Figures and Tables

**Table 1:** Summary of the main methods for establishing 3D models for drug screening.

Type of 3D Technique	Name of the Technique	Endpoint Assay and Data Acquisition	Ref.
Scaffold-free	Hanging drop	Viability/Cytotoxicity: CellTiter-Glo^®^ 3D, LIVE/DEAD (Calcein AM/ethidium homodimer); Trypan blue; Perfecta3D^®^; Other Analysis: WB, IHC, IF and LS-FM.	[31,32]
	Forced floating (e.g., Ultra-low attachment plates)	Viability/Cytotoxicity: CellTiter-Glo™ 3D, LIVE/DEAD (Calcein AM/ethidium homodimer); ViaLight™ Plus. Other Analysis: WB, qPCR, IF, IHC, HCI (Software: Cytation 3, CellInsight NXT, MetaXpress 6).	[31,55,56,57,58]
	Micromolding	Viability/Cytotoxicity: LIVE/DEAD (Calcein AM/propidium iodide), CCK-8, MTT. Other Analysis: WB, qPCR, Flow Cytometry, Hematoxylin and eosin staining.	[59,60]
	Agitation-based techniques	Viability/Cytotoxicity: CellTiter-Glo^®^ 3D; LIVE/DEAD (Calcein AM/ethidium homodimer); Trypan blue; Perfecta3D^®^. Other Analysis: IF and LS-FM.	[32]
	Magnetic levitation or bioprinting	Viability/Cytotoxicity: CellTiter-Glo^®^ 3D; LIVE/DEAD (Calcein AM/ethidium homodimer); Trypan blue; Perfecta3D^®^. Other Analysis: Reporter transgene, IF, LS-FM, ELISA.	[32,61,62,63]
	Microfluidics	Viability/Cytotoxicity: LIVE/DEAD (Calcein AM/ethidium homodimer); Calcein AM (LIVE) and 7-Amino-ActinomycinD (DEAD) staining Other Analysis: Flow Cytometry, SEM, PCM, Reporter transgene, IF, qPCR, Actin Cytoskeleton and Focal Adhesion Staining Kit	[64,65,66]
	Pellet Culture	Viability/Cytotoxicity: CellTiter-Glo^®^ 3D; LIVE/DEAD (Calcein AM/ethidium homodimer), Trypan blue; Perfecta3D^®^; Other Analysis: IF and LS-FM.	[32]
Scaffold-based	3D-bioprinting	Viability/Cytotoxicity: LIVE/DEAD (Calcein AM/propidium iodide); Alamar Blue, CCK-8, LDH. Other Analysis: MMP Zymography Assay Kit (for matrix metalloproteinase characterization), SEM, Histology, IHC, IF, qPCR.	[34,67,68,69]
	Microfluidics	Viability/Cytotoxicity: LIVE/DEAD (Calcein AM/ethidium homodimer), CCK-8. Other Analysis: IF, MMP Zymography Assay Kit, FACS, Caspase 3/7 activity assay, CellTrace™ CFSE Cell Proliferation Kit	[69,70]
	Hydrogel	Viability/Cytotoxicity: CellTiter-Glo^®^ 3D, LIVE/DEAD (Calcein AM/ethidium homodimer); Other Analysis: qPCR, IF	[58,71]

CCK-8: Cell Counting kit-8 cell proliferation assay; ELISA: Enzyme-Linked Immunosorbent Assay; FACS: Fluorescence-Activated Cell Sorting; HCI: High-Content Imaging; IF: Immunofluorescence; IHC: Immunohistochemistry; LDH: Lactate Dehydrogenase; LS-FM: Light-Sheet Fluorescence Microscopy; qPCR: Quantitative Real-time Polymerase Chain Reaction; PCM: Phase-Contrast Microscopy; SEM: Scanning Electron Microscopy; WB: Western Blotting.
